# Early Neolithic pastoral land use at Alsónyék-Bátaszék, Hungary (Starčevo culture): New insights from stable isotope ratios

**DOI:** 10.1371/journal.pone.0295769

**Published:** 2023-12-12

**Authors:** Magdalena Blanz, Marie Balasse, Delphine Frémondeau, Erika Gál, Anett Osztás, Anna Zs. Biller, Éva Á. Nyerges, Denis Fiorillo, Eszter Bánffy, Maria Ivanova

**Affiliations:** 1 Vienna Institute of Archaeological Science (VIAS), University of Vienna, Vienna, Austria; 2 Human Evolution and Archaeological Sciences (HEAS), University of Vienna, Vienna, Austria; 3 AASPE ‘Archéozoologie, Archéobotanique: Sociétés, Pratiques et Environnements’, CNRS/MNHN, Paris, France; 4 Centre for Archaeological Sciences, University of Leuven, Leuven, Belgium; 5 Department of Archaeology, University of Reading, Reading, United Kingdom; 6 Institute of Archaeology, Research Centre for the Humanities, Eötvös Loránd Research Network, Budapest, Hungary; 7 Aquincum Museum, Budapest History Museum, Budapest, Hungary; 8 Department of Archaeology, Savaria Museum, Szombathely, Hungary; 9 Römisch-Germanische Kommission des Deutschen Archäologischen Instituts, Frankfurt am Main, Germany; 10 Vor- und Frühgeschichtliche Archäologie, Johannes Gutenberg-Universität Mainz, Mainz, Germany; University of Edinburgh, UNITED KINGDOM

## Abstract

The earliest introduction of livestock (cattle, goats, sheep, pigs) into the Carpathian Basin was an important step towards farming expansion into continental Europe. This spread beyond the environments of the southern Balkans was accompanied by a reduction in the spectrum of cultivated crops, changes in the relative representation of different domestic animals, and, most likely, adaptations of husbandry practices. How the earliest farmers in the Carpathian Basin kept their domestic stock is still understudied. We explored early animal management and land use strategies at the Starčevo settlement at Alsónyék-Bátaszék, Hungary (Early Neolithic, ca. 5800–5600 cal BC). Settled at the intersection of wide alluvial plains, waterlogged meadows and marshes to the east, and forested hills to the west, early farmers at Alsónyék had a wide variety of options for nourishing their livestock. We performed stable isotope ratio analysis of bone collagen (n = 99; δ^13^C, δ^15^N) and tooth enamel (n_teeth_ = 28, sequentially sampled for δ^13^C and δ^18^O) from wild and domestic animals to locate them in the landscape and investigate herding practices on a seasonal scale. The bone collagen isotope ratios mostly indicate feeding in open environments. However, results from the sequential analysis of cattle and sheep enamel suggest diverse dietary strategies for winters, including consumption of forest resources, consumption of summer hay and grazing in an open environment. Most pigs appear to have had herbivorous diets, but several individuals likely supplemented their diet with animal protein. Stable isotope ratio results from the Lengyel phase at Alsónyék (ca. 4800–4300 cal BC) suggest more access to animal protein for pigs, and feeding in more open areas by wild boar, red deer and cattle compared to the Starčevo phase. This study’s results demonstrate considerable variability in early animal husbandry practices at Alsónyék.

## Introduction

Domesticated animals and plants, initially originating from Southwest Asia and domesticated in the 10^th^-9^th^ millennium BC [1], were introduced by Neolithic farmers to Thessaly and Aegean Macedonia in the early 7^th^ millennium BC. The spread of agriculture to inland Europe took a decisive step at the turn of the 7^th^ to the 6^th^ millennium BC when settlers from the Starčevo-Çris-Körös cultural complex pioneered the northern Balkans and the Southern Carpathian Basin (modern northern Bulgaria, Serbia, Romania and Hungary; [2–4]). This northward diffusion of farming was accompanied by significant changes in the spectrum of plants cultivated and the species composition of animals kept [5–8]. In the sub-Mediterranean southern Balkans (modern-day Northern Greece and southern Bulgaria), a broad spectrum of crops was cultivated [8], which appears to have been the result of a strategy of diversification. Correlated with differences in environments and climatic conditions, expansion into the northern Balkans and the Great Hungarian Plain was generally accompanied by a reduction in the spectrum of crops cultivated, and the proportion of sheep, goats, and pigs diminished while cattle became dominant in many faunal assemblages (reviewed in [9]). Early Neolithic farmers in the Great Hungarian Plain settled preferentially on flat surfaces on hydromorphic meadow soils near freshwater [10]. Living in an environment of marshland islets and forested hills likely encouraged a large degree of adaptation by these early farmers [11]. Further investigations are needed to describe in more detail how these farming systems have adapted to the advantages and constraints of the landscape, including in terms of the respective–and not necessarily exclusive–spatial locations of crops and livestock at the site scale, as well as on a seasonal scale.

**Fig 1:**
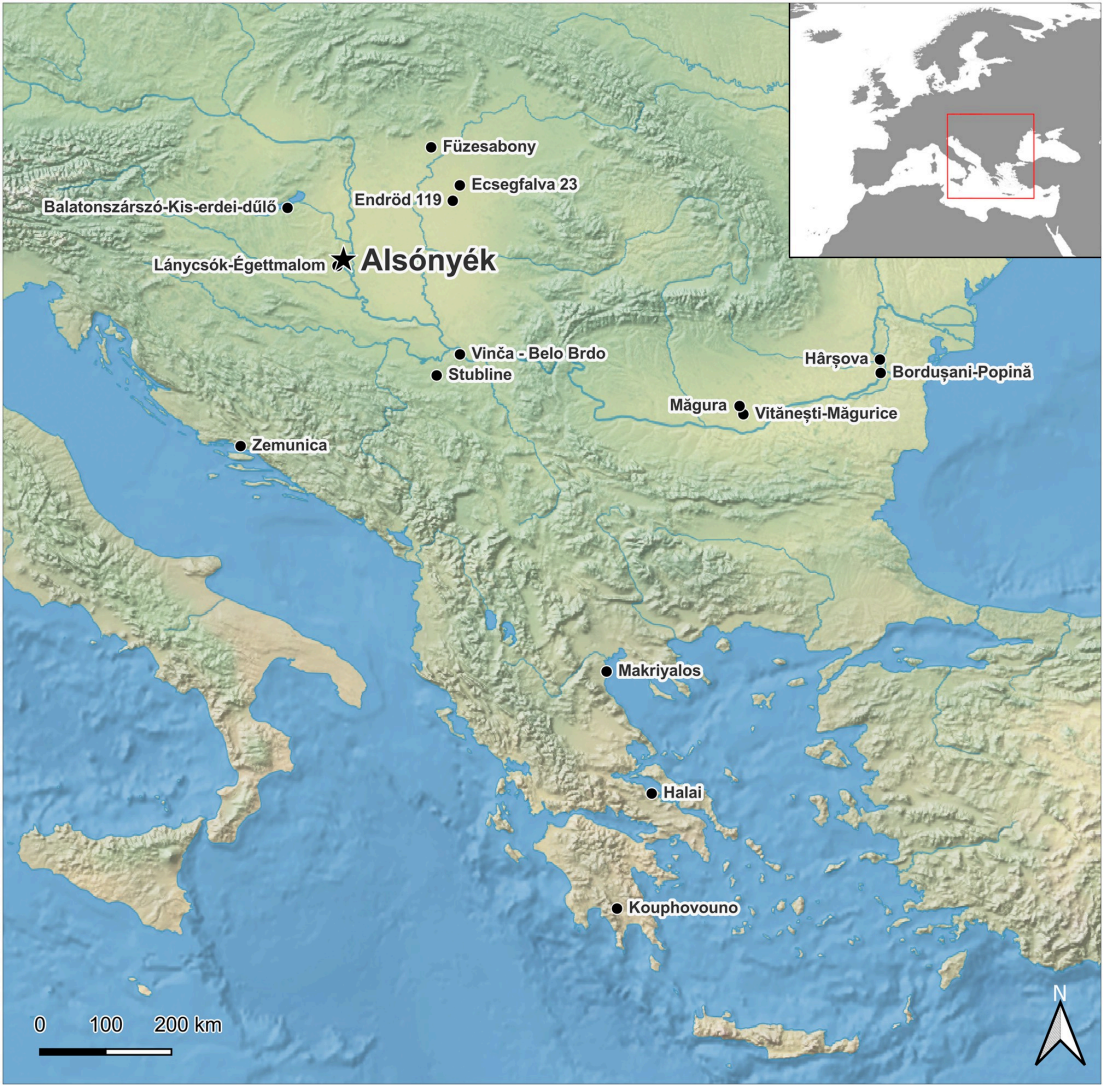
Map showing the location of Alsónyék and other sites mentioned in this article. Map data: Natural Earth II from www.NaturalEarthData.com (public domain).

This study focuses on animal husbandry at Alsónyék-Bátaszék in southern Hungary (Fig 1; Lat. 46° 12’ N, Long. 18° 42’ E), which is particularly suited to such investigations of the place of animals in the landscape due to being located at an intersection of different ecosystems. Using stable isotope ratios (δ^13^C, δ^18^O, δ^15^N) of animal teeth and bones, the aim of this study is to research in what environments cattle and sheep (the dominant species) were herded and how pigs–rare but present–were kept in the Early Neolithic, to understand how herders utilised Alsónyék’s wider surrounding environment. Analysis of sequential tooth enamel samples also allows investigations of diets on a seasonal scale, through which it is possible to examine the use of woodlands and wetlands as potential seasonal food sources, as well as the proximity of pigs to dwellings. To construct a framework for the range of possibilities in animal husbandry practices, references are also made to ethnographic and historical accounts from Hungary from the 18^th^ to 20^th^ centuries. This work contributes to more wide-ranging research on understanding the environmental, biological and socio-cultural factors in the initial dispersal and adaptation of farming systems across Europe.

### Alsónyék: Description of the site, environment, and subsistence practices

Alsónyék subsite 5603/1 (see Fig 38 in [12]), where most of the Starčevo features at Alsónyék were found, was occupied by people from the Starčevo cultural complex in the first half of the 6^th^ millennium cal BC (beginning ca. 5800–5730 cal BC, and ending 5575–5505 cal BC, both 95% probability [13]). It appears as a substantial, clustered settlement [14]. Later occupation of the site included Neolithic communities from the Linearbandkeramik (LBK), Sopot and Lengyel cultures (Table 1). The village lies in the Carpathian Basin in the southwestern part of Tolna Sárköz in Southeast Transdanubia (see Fig 1) at the transition of hilly margins to marshy lowlands [15].

**Table 1:** Sites mentioned in the text with their associated cultural groups and phases as relevant to this study.

Site name	Cultural group	Chronological phase	Dating cal BC	Reference
Alsónyék	Starčevo	Early Neolithic	ca. 5800–5600	this study
	LBK	Middle Neolithic	ca. 5500–5000	[25]
	Sopot	Middle/Late Neolithic	ca. 5100–4800	[25]
	Lengyel	Late Neolithic	ca. 4800–4300	[25]
Balatonszárszó	Early LBK	Middle Neolithic	ca. 5450–5250	[26]
Vinča-Belo Brdo	Vinča-Pločnik II	Late Neolithic	ca. 4850–4600	[27]
Borduşani-Popină	Gumelniţa A2	Late Chalcolithic	ca. 4500–4250	[28]
Ecsegfalva	Körös	Early Neolithic	ca. 5800–5600	[29]
Endrőd 119	Körös	Early Neolithic	ca. 5800–5630	[30]
Füzesabony	ALP	Middle Neolithic	ca. 5550–5000	[26]
Halai	-	Early Neolithic	ca. 6400–5800	[31]
Hârşova-tell	Gumelniţa A2	Chalcolithic	ca. 4350–4000	[28]
Kouphovouno	-	Middle Neolithic	ca. 5800–5400	[31]
Lánycsók-Égettmalom	Starčevo	Early Neolithic	ca. 5800–5600	[32]
Măgura—Boldul lui Moş Ivčnus	Starčevo-Criş I	Early Neolithic	ca. 6000–5800	[33]
Makriyalos II	-	Late Neolithic II	ca. 4950–4500	[31]
Stubline	Vinča-Pločnik II	Late Neolithic	ca. 4850–4600	[27]
Vităneşti-Măgurice	Gumelniţa A2	Chalcolithic	ca. 4450–4150	[28]
Zemunica	Impressed Pottery	Early Neolithic	ca. 6000–5800	[34]

References are to relevant studies on faunal stable isotope ratios (δ^13^C and δ^15^N). ALP = Alföld Linear Pottery, LBK = Linearbandkeramik.

The Danube is 16 km to the east of Alsónyék-Bátaszék (hereafter: Alsónyék) today, and, together with its tributary Sárvíz, it shaped the landscape considerably [15]. Former Danube channels left behind oxbow lakes and wide alluvial floodplains, dominated by (seasonally) waterlogged meadows and marshes, likely frequently inundated, and island-like plateaus above the floodplain [16, 17]. Gallery woods were likely present along rivers and oxbow lakes, and groves may have grown in the floodplains [18]. The settlement site of Alsónyék itself appears to have been outside the extensive flooding area [16], but compared to later groups, the Starčevo community at Alsónyék occupied the lower and wetter parts of the area [19]. Although crop cultivation to the east of the site was likely possible on the elevated islets dotted throughout the landscape, it would have been at high risk of harvest-loss due to flooding, likely making the cultivation of arable land to the west of the site more favourable [16].

To the west of Alsónyék, the Danubian floodplains are supplanted by skeletal soils and forested hills (Szekszárd Hills, 273 m; [14, 16]), which also provided arable land on the hilltops [16]. An open oak woodland would likely have been present which may have also included pine, elm, hazel, lime, beech and hornbeam [20]. Wild fauna during the Starčevo phase includes animal species consistent with both forested and open wetland environments, e.g. wildcats (*Felis silvestris*), beavers (*Castor fiber*) and multiple duck species (*Anatinae*) [21–23].

The faunal assemblage from the Starčevo phase at subsite 5603/1 in the southeastern part of Alsónyék is dominated by domestic animals (88%), with caprines (sheep and goats; for scientific names see Tables 2 and 3) and cattle being predominant, and only minor amounts of pigs (3%) and dogs (Table 2; data given as number of identifiable specimens (NISP) from [23]). Most cattle have been slaughtered as juveniles and adults, consistent with a focus on dairying, while pigs were mostly slaughtered as juveniles [23]. Hunting was not a dominant subsistence activity, with only ca. 12% of the identifiable mammal faunal assemblage being wild animals, mostly red deer and wild boar [23]. Freshwater fish remains were also present at the site (1.4% of NISP_faunal remains_), including wels catfish, northern pike and carp [20, 21].

**Table 2:** Mammal faunal spectrum at Alsónyék subsite 5603/1 from the Starčevo phase (identifiable mammals only).

Species	Common name	NISP	% of NISP_identifiable mammal_
*Ovis aries/Capra hircus* *	sheep/goat*	4688	42.5
*Bos taurus*	cattle	4633	42.0
*Sus domesticus*	pig	331	3.0
*Canis familiaris*	dog	46	0.4
*Cervus elaphus*	red deer	565	5.1
*Sus scrofa*	wild boar	525	4.8
*Capreolus capreolus*	roe deer	160	1.5
other (identifiable)		78	0.7
**Total**		**11026**	**100**

*Around six sheep per goat; data from [23].

**Table 3:** Bone collagen δ^13^C and δ^15^N stable isotope ratios from the Starčevo phase at Alsónyék, subsite 5603/1.

Species	common name	n	δ^13^C (‰)			δ^15^N (‰)		
			Min.	Median	Max.	Min.	Median	Max.
*Bos taurus*	cattle	10	−21.55	−20.71	−19.44	4.96	5.41	7.07
*Ovis aries*	sheep	18	−21.62	−20.76	−19.95	4.41	6.82	7.45
*Sus domesticus*	pig	13	−22.36	−20.43	−19.13	6.05	6.89	9.16
*Sus scrofa*	wild boar	19	−22.11	−21.08	−19.89	3.95	6.99	8.59
*Canis familiaris*	dog	6	−22.54	−21.27	−20.72	6.05	8.82	9.43
*Cervus elaphus*	red deer	10	−22.55	−21.46	−19.79	4.27	6.63	7.76
*Capreolus capreolus*	roe deer	10	−23.72	−21.20	−20.14	5.47	6.36	7.14
*Vulpes vulpes*	fox	2	−20.35	−19.64	−18.93	7.70	7.79	7.87
*Abramis brama*	bream	2	−24.37	−23.69	−23.01	8.00	8.63	9.25
*Cyprinus carpio*	carp	3	−27.18	−27.15	−25.97	6.86	8.37	8.64
*Esox lucius*	northern pike	2	−22.11	−21.41	−20.70	9.97	9.98	9.98
*Sander lucioperca*	zander	3	−21.77	−20.15	−19.13	9.47	9.81	10.18
*Silurus glanis*	wels catfish	1	−20.64	−20.64	−20.64	8.24	8.24	8.24

For additional information see Table A in S1 File; sample numbers exclude samples that failed the quality criteria.

The recovered crops from the Starčevo phase at Alsónyék (einkorn, emmer and barley [24]) indicate that at this time a mixed farming economy was likely present, and people were somewhat locally bound due to the requirements of crop cultivation. Livestock herding could have taken place in the forested hills to the west of the site, in the wetlands and waterlogged humid meadows in the east along the river Sárvíz (pigs in particular; [16]), or on cropland after harvest.

### Stable isotope ratios for palaeodietary reconstruction

#### Bone collagen

Bone collagen stable carbon (δ^13^C) and nitrogen (δ^15^N) isotope ratios reflect those of consumed dietary protein (and other dietary components to a lesser extent) in the last years of an individual’s life with a systematic diet-collagen offset (ca. 5–6‰ for δ^13^C, and ca. 3–5‰ for δ^15^N; [35–40]). Therefore, bone collagen δ^13^C and δ^15^N values can be used to determine what food groups were commonly consumed. Consumer δ^15^N values are frequently used as indicators of trophic level and can be used to infer e.g. whether pig diets were likely supplemented with animal protein [28, 41–43], or consisted of fertilised crops [44], both elevating δ^15^N values. In younger individuals, milk consumption puts the offspring at a higher trophic level than the mother leading to higher δ^15^N values. However, when growth rates are high (e.g. in adolescence), δ^15^N values tend to be lower [45]. This introduces some uncertainties in interpretations of δ^15^N values of young animals.

Hungary’s terrestrial vegetation is dominated by C_3_-plants, with only minor amounts of C_4_ plants being present in dry grasslands following recent colonisations [46], so that C_4_ plants were likely not contributing to Early Neolithic diets at Alsónyék to a significant extent. Forest grazing/browsing tends to lead to lower consumer δ^13^C values due to the canopy effect [47, 48]. Ground-level undergrowth was found to be depleted by 2–5‰ in dense forest compared to plants and trees in open environments [47, 49]. Bone collagen δ^13^C values from modern forest-dwelling red and roe deer from dense deciduous forests in Dourdan (France) and Białowieża (Poland) were on average −23.7‰, with all except one below −22.5‰ (n = 29; corrected for the fossil fuel effect; [48]). Values approaching these thresholds are therefore considered to likely reflect a significant dietary contribution of forest resources (particularly ground-level undergrowth), especially in the case of herbivores. However, not all animals occupying forests necessarily have low δ^13^C values, possibly in part due to feeding outside of the forest [50] and feeding on fruits (e.g. acorns) which are higher in δ^13^C, and can lead to higher δ^13^C values than expected for forest-dwelling animals, particularly in the case of suids [51]. Conversely, low δ^13^C values in suids may also occur due to consumption of freshwater fish [28].

Since water stress has been shown to elevate δ^13^C values [52], and heavy watering leads to around 1‰ lower δ^13^C values [53–55], it has been argued that freshwater wetlands may be expected to have lower δ^13^C values than C_3_ vegetation from drier areas [49, 56], lowering consumer δ^13^C values. However, compared to e.g. the canopy effect, this is likely only a minor effect.

Since δ^13^C and δ^15^N values differ due to a large variety of different factors, it is important to establish site-specific baselines for different diets. To gain comparative references of the typical δ^13^C and δ^15^N values for the diets of the domesticated animals under study (cattle, pig, sheep), bones of wild fauna like wild boar, red and roe deer (primarily forest-dwelling), freshwater fish (potentially consumed by pigs), as well as dogs (consumers of higher trophic level foods) excavated from the same site and phase are also analysed.

### Sequential analysis of tooth enamel

Sequential analysis of stable carbon and oxygen isotope ratios in tooth enamel gives access to dietary information on a seasonal scale. Bioapatite precipitates in oxygen isotopic equilibrium with body water [57], linked, through the ingestion of surface water, to local annual precipitation [58, 59], whose δ^18^O values vary seasonally with air temperature in continental Europe [60]. Additionally, animal behavior and physiology in response to seasonal changes in temperature and air humidity also affect body water oxygen isotope composition [61]. All factors combine to create a seasonal signal in tooth enamel δ^18^O values. Sequential sampling permits the retrieval of a one-year record from the third molar of sheep and cattle [62], or by combining measurements from the first and second incisors in suids [51], while the canines of male suids may provide a pluriannual record [51].

The stable carbon isotope ratios in bioapatite reflect those of the entire diet [35] as opposed to collagen, whose primary carbon source is proteins [39, 63]. A 14.1‰ isotope enrichment (ε) has been shown between diet and enamel bioapatite δ^13^C values in a variety of large ruminant mammals [64], while this enrichment is 13.3‰ in experimental pigs fed C_3_ diets [65]. Most C_3_ plants have δ^13^C values varying from −29‰ to −25‰ around an average value of −27‰ in open areas [66], or −25.5‰ in preindustrial times after correction for the fossil fuel effect [67]. This should lead to an average value of −11.8‰ in sheep and cattle enamel bioapatite, or −12.5‰ in pig enamel bioapatite–although a significant fruit component in suid diets [68, 69] would also elevate this value; the δ^13^C values measured in predominantly open areas and under continental climate are globally comprised between −13‰ and −9‰ in cattle and sheep tooth enamel [33, 70]. By contrast, animals dwelling in closed forests have lower bone collagen δ^13^C values (below −22.5‰, [48]), so that enamel bioapatite δ^13^C values should tend towards –13,8‰ in large ruminants (applying a 5% spacing between collagen and diet [39] and a 14.1‰ isotope enrichment between diet and enamel bioapatite [64]), or towards −14.6‰ in pigs (applying a 13.3‰ isotope enrichment between diet and enamel bioapatite [65]) although a significant contribution of forest fruits would elevate this value. We consider values approaching these thresholds as reflecting a significant contribution of forest resources to animal diet.

Seasonal variations are expected in plant δ^13^C values, in response to seasonal changes in the environmental factors affecting the stomatal aperture and therefore the carbon isotope discrimination during photosynthesis, and also possibly due to changes in plant physiology during the growing season. The highest δ^13^C values are expected in the summer when the air temperature is the highest and air humidity and ground water are the lowest [71–73]. Consequently, δ^18^O and δ^13^C values are expected to follow the same pattern of seasonal variation. Indeed, in modern sheep grazing on the same pasture throughout the year, the δ^18^O and δ^13^C sequences in enamel were shown to vary in phase or very close to it [74] with amplitudes of variation of 1 to 3‰ in δ^13^C [75]. Deviations from this pattern may result from the contribution of different food sources during the year due to foddering or mobility between areas where environmental factors affect plants differently.

## Materials and methods

### Bone collagen stable isotope ratio analysis

All bone and tooth samples in this study were excavated from Alsónyék subsite 5603/1, and date to the Early Neolithic, Starčevo phase (ca. 5800–5600 *cal* BC; [13]; stored at the Wosinsky Mór County Museum, Szekszárd). Around 2 g cortical bone samples of long bones were taken from domestic and wild terrestrial and aquatic fauna (n = 107; Table 3). Differentiation between wild boar and domestic pig was done according to size, following [76]. In the case of the mammals, the same part of the bone from the same side of the body (within each species group) was preferentially sampled, to prevent sampling the same individual twice. To enable this sampling strategy, in some cases, juvenile and subadult individuals were also sampled (see Table A in S1 File). This needs to be taken into account when interpreting the results as it introduces uncertainty when comparing between animals of differing ages, e.g. due to different nitrogen balances (see above). In the case of pigs, most samples were from younger individuals because pigs tend to be slaughtered as juveniles/subadults and few adult pig bones were available to sample. Previous studies have argued for including younger livestock in stable isotope ratio analyses (e.g. [77]) since this enables a more complete insight into husbandry practices without a bias toward adults.

Bone samples were cleaned by abrading the outer surface using a tungsten carbide drill bit. Collagen was extracted from 200–230 mg ground bone using 1 M HCl, followed by 0.125 M NaOH, following a modified Longin (1971) method [78] described in [79], but with the gelatinisation step at 70 °C instead of 100 °C. In the case of fish bones, the NaOH step was shortened to initially 15 minutes. If the solution coloured during this time, it was exchanged for fresh 0.125 M NaOH solution for another 15-minute immersion (following [80]).

Using an elemental analyser (EA; Thermo Flash 2000) interfaced with an isotope ratio mass spectrometer (IRMS, Thermo DeltaVAdvantage), coupled δ^13^C and δ^15^N measurements of 320-380 µg bone collagen were conducted. Within each run, multiple replicates of a secondary alanine standard were included (calibrated to primary standards IAEA-600 for δ^13^C, and IAEA-USGS25, IAEA-N-1 and IAEA-N-2 for δ^15^N). The alanine standards gave mean values of −21.47±0.09‰ for δ^13^C (mean±σ; expected value: −22.16‰ with reference to VPDB) and 0.70±0.13% for δ^15^N (expected value: +0.59‰ with reference to AIR), and 40.07±1.46% for C content (expected value: 40.44%) and 15.56±0.09% for N content (expected value: 15.72%) over the course of all measurements (n = 23), and were used to correct the measured data. Quality criteria to ensure data robustness were chosen such that results were rejected if the bone collagen yield was less than 1%, or if the collagen had a C/N (molar) ratio outside of 2.9–3.45, or if the C content was less than 13%, or the N content less than 4.8% (following suggestions in [81–83]).

Isotopic niche spaces were modelled as Bayesian ellipses with the R package SIBER [84].

### Tooth enamel sequential stable isotope ratio analysis

The study material includes teeth from cattle (11 upper third molars-M3), sheep (9 lower M3), pig (1 lower M3), red deer (2 lower M3) and wild boar (1 upper canine, 1 upper M3, 1 lower M3, 2 lower incisors from the same individual; Table C in S2 File). The enamel surface was cleaned with a tungsten carbide drill bit. Teeth were sequentially sampled using a diamond-coated drill bit on the lingual side of the anterior lobe for the cattle M3; on the vestibular side of the middle lobe for the sheep and red deer M3; on the lingual side of the anterior cusp for the suid molars, and on the labial side for the suid incisors and the maxillary canine. The sampling procedure for the suid teeth was illustrated in [51]. These enamel powders were pre-treated to eliminate diagenetic carbonates (0.1 M acetic acid for 4 h at room temperature, 0.1 ml/mg). Pre-treated enamel samples weighing ~600 µg were reacted with 100% phosphoric acid at 70°C in individual vessels in an automated cryogenic distillation system (Kiel IV device), interfaced with a DeltaVAdvantage isotope ratio mass spectrometer. The analytical precision for each run, estimated from 5 to 8 analyses of our laboratory carbonate standard (Marbre LM, calibrated to the NBS-19 international standard) was always less than or equal to 0.05‰ for δ^13^C values and 0.04‰ for δ^18^O values (both with reference to VPDB). For each run, the Marbre LM gave a mean δ^13^C value comprised between 2.09‰ and 2.20‰ (expected value 2.13‰) and a mean δ^18^O value comprised between −2.02‰ and −1.96‰ (expected value −1.83‰). The δ^18^O values for sheep and cattle tooth enamel were previously published in [85, 86] respectively. In the sheep and cattle molars and in the wild boar canine, showing full annual cycles, the phase shift between the δ^18^O and δ^13^C sequences was determined using a sinusoidal model approach after [74]; see also Table H in S2 File.

## Results

### Bone collagen stable isotope ratios

**Fig 2:**
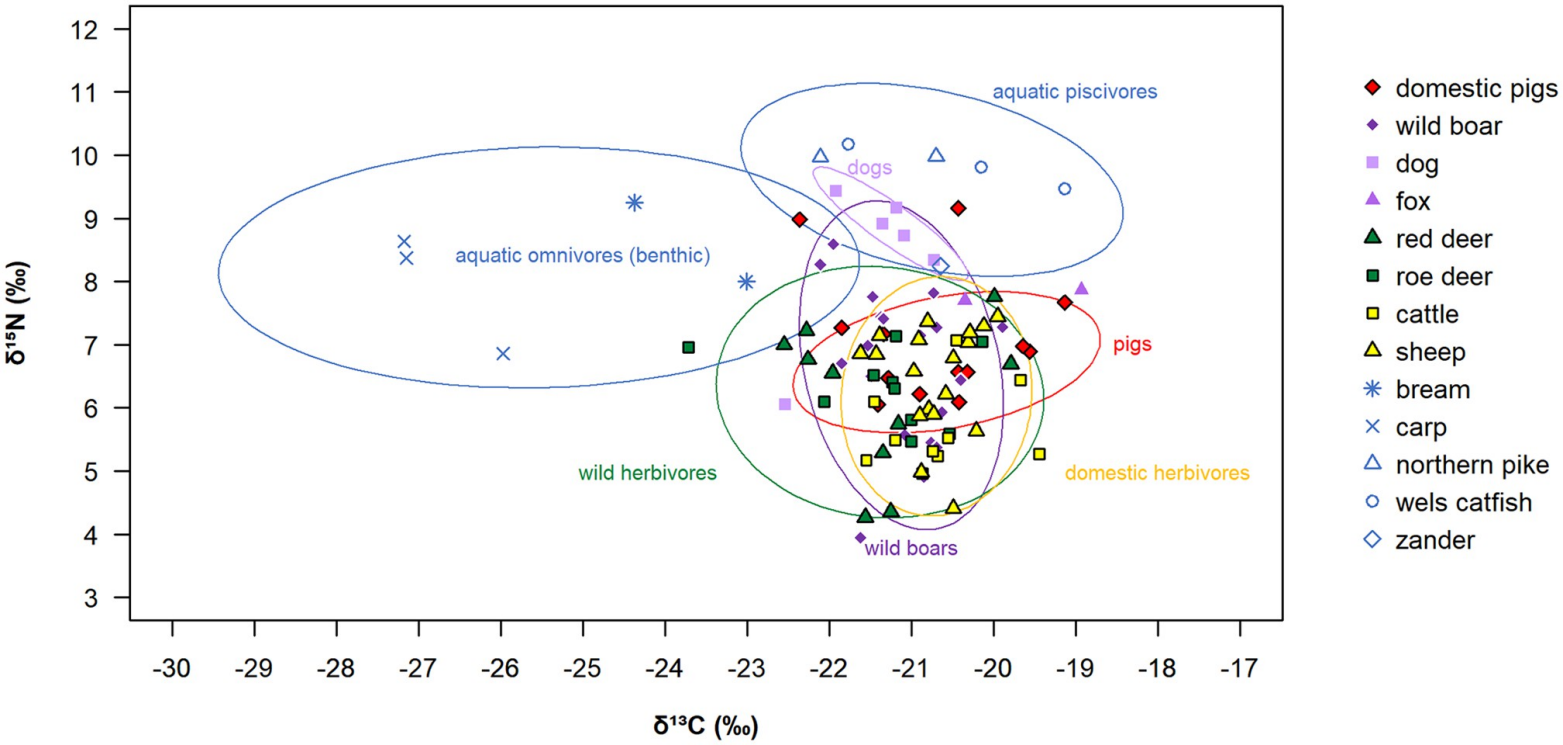
Stable carbon (δ^13^C) and nitrogen (δ^15^N) isotope ratio results for bone collagen from the Starčevo phase at Alsónyék, subsite 5603/1. The 90% prediction ellipses were modelled excluding an outlier dog and two pig datapoints (see S3 File).

**Fig 3:**
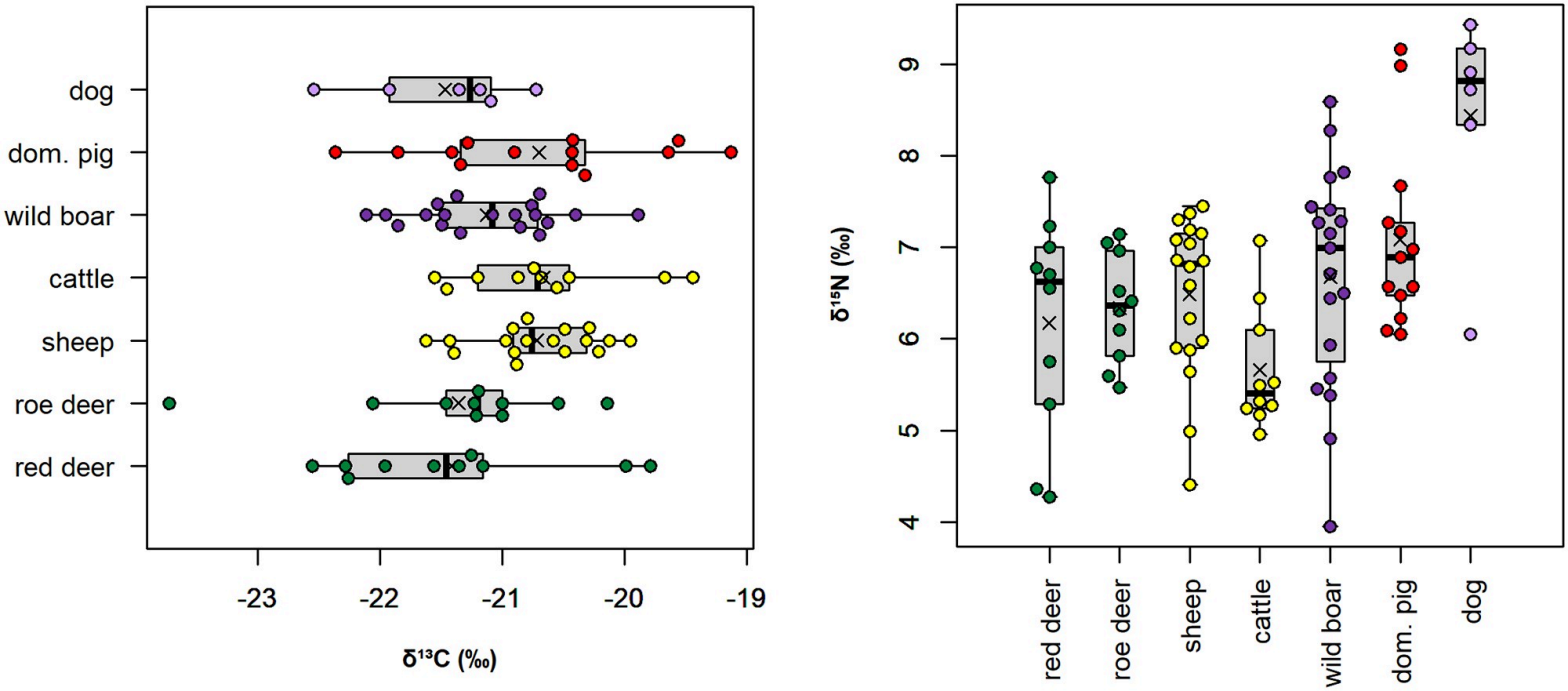
Stable carbon (δ^13^C) and nitrogen (δ^15^N) isotope ratio results for mammal bone collagen from the Starčevo phase at Alsónyék, subsite 5603/1. This figure shows the same data as in Fig 2, but visualised as boxplots. The line in the boxplot indicates the median, and the cross the mean. Whisker boundaries were chosen as 1.5 × interquartile range. Fish and foxes were excluded due to low sample numbers.

During collagen extraction, four fish bone samples dissolved completely and could not be analysed, and the measured results from one sheep and four fish were excluded because they did not meet the quality criteria (see Table A in S1 File, where the complete set of data is also listed). Table 3 and Figs 2 and 3 show the δ^13^C and δ^15^N results for the samples that passed the quality criteria.

The results for most species overlap to a large extent (Figs 2 and 3). A one-way analysis of variance (ANOVA) was conducted to compare δ^13^C values between pigs, wild boar, dogs, red deer, roe deer, cattle and sheep (F(6,79) = 2.53, p = 0.027). It revealed differences in the mean δ^13^C values between species, but a post-hoc Tukey test showed no significant differences. A second one-way ANOVA (F(6,79) = 5.81, p = 0.00005) followed by a Tukey test indicated differences in mean δ^15^N values between pigs and cattle, and between dogs and wild boar, red deer, roe deer, cattle and sheep. Domestic pigs had a smaller range of δ^15^N values than wild boar (3.1‰ compared to 4.6‰; Fig 3, right). There was no statistically significant difference in δ^15^N or δ^13^C between juvenile (n = 6) and subadult pigs (n = 6), and no other age-based differences were identified.

Red deer and roe deer yielded on average the lowest δ^13^C values among terrestrial mammals (Fig 3), the lowest collagen δ^13^C value being measured in a roe deer (−23.7‰, ALSCap9), followed by three red deer with δ^13^C values between −23.0‰ and −22.0‰, which are close to and below the threshold value suggesting a significant contribution of forest resources to the animals’ diets. The sampled freshwater fish exhibited a wide breadth of δ^15^N and δ^13^C values, with differences visible between dietary groups: benthic omnivores like carp and bream had the lowest δ^13^C values (−27.2‰ to −23.0‰) of all measured samples, whereas piscivorous fish (northern pike, wels catfish and zander) had the highest δ^15^N values (8.2‰ to 10.2‰).

### Tooth enamel sequential stable isotope ratios

**Fig 4:**
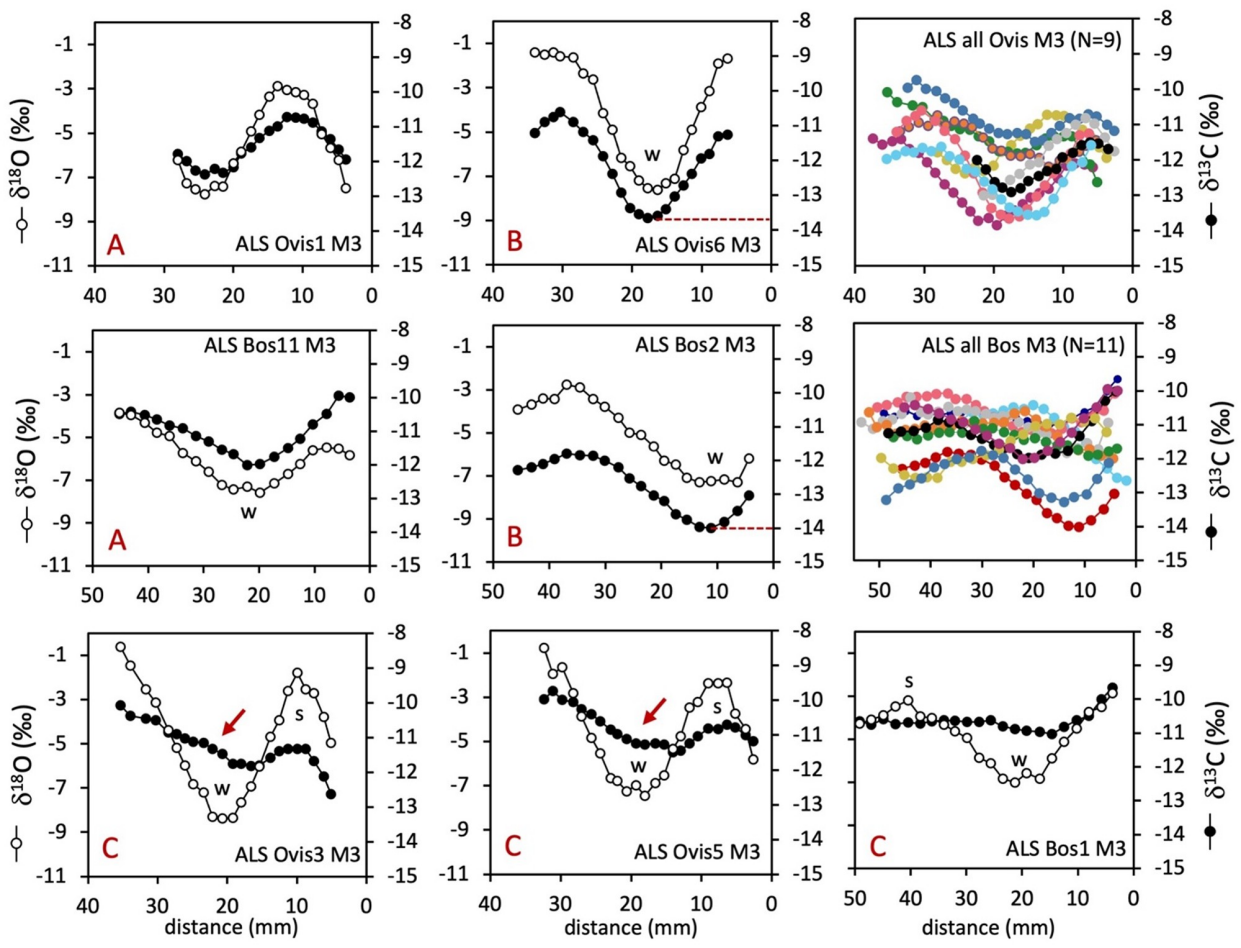
Results from the sequential analysis of stable carbon (δ^13^C) and oxygen (δ^18^O) isotope ratios in cattle (Bos) and sheep (Ovis) third molars from the Starčevo phase at Alsónyék (ALS). The figure shows some typical results for the different observed patterns. Patterns A, B and C: see main text. W = winter; S = summer.

**Fig 5:**
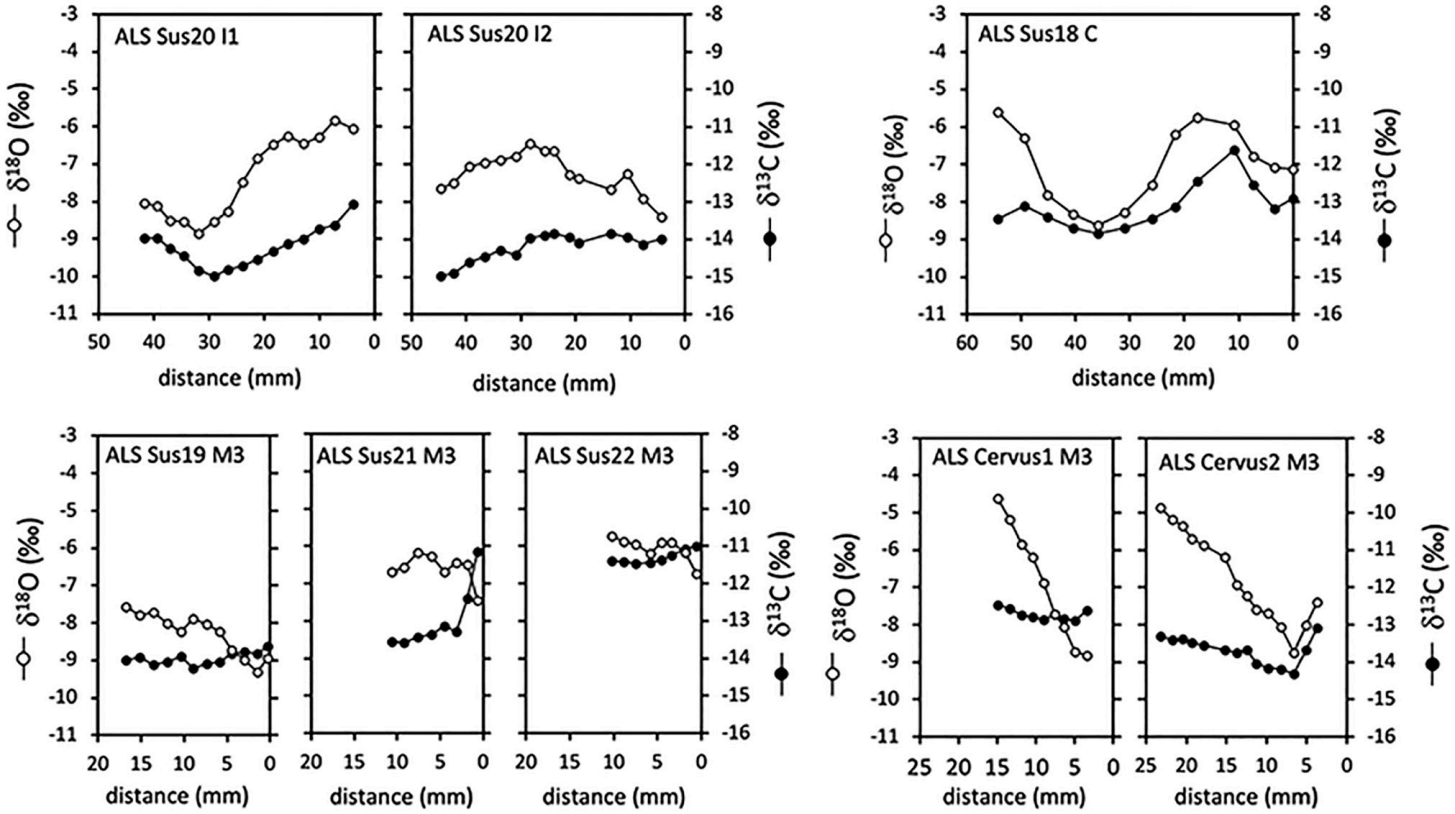
Results from the sequential analysis of stable carbon (δ^13^C) and oxygen (δ^18^O) isotope ratios in tooth enamel. Samples were wild boar (Sus18, 19, 20 and 21), pig (Sus22) and red deer (Cervus1 and 2) from the Starčevo phase at Alsónyék.

The results from the sequential analysis of tooth enamel are shown in Figs 4 and 5, Table 4, and Tables D-H in the S2 File (including phase shift modeling). Sheep enamel bioapatite δ^13^C values vary overall between −13.8‰ and −10.0‰. All sheep have recorded seasonal changes in their diet δ^13^C values (Fig 4). The amplitude of intra-tooth variation varies between 1.5‰ (ALS Ovis4) and 3.5‰ (ALS Ovis6). Different patterns of variations are observed. Most sheep show a sinusoidal variation in δ^13^C values in phase with the seasonal changes in δ^18^O values (phase shift comprised between 331 and 368°, Table H in S2 File; Patterns A and B in Fig 4). Among those, three sheep (ALS Ovis2, Ovis6 and Ovis9) have lower winter δ^13^C values (−13.8‰ to −13.6‰) tending towards the threshold indicating significant consumption of forest resources (Figs 4 and 5, Pattern B). ALS Ovis3 and ALS Ovis5 deviate from the sinusoidal pattern of variation and have a reduced amplitude of variation between δ^13^C values recorded in winter and the consecutive summer (0.1‰ and 0.5‰ respectively; Pattern C, Fig 4).

**Table 4:** Tooth enamel carbon and oxygen stable isotope ratios from the Starčevo phase at Alsónyék.

Specimen	common name	Species	δ^13^C (‰)				δ^18^O(‰)			
			Min	Max	M	Δ	Min	Max	M	Δ
ALS Cervus1 M3	red deer	*Cervus elaphus*	-12.9	-12.5	-12.7	0.4	-8.8	-4.6	-6.7	4.2
ALS Cervus2 M3	red deer	*Cervus elaphus*	-14.3	-13.1	-13.7	1.2	-8.7	-4.9	-6.8	3.9
ALS Sus20 I1	wild boar	*Sus scrofa*	-15.0	-13.1	-14.0	1.9	-8.8	-5.8	-7.3	3.0
ALS Sus20 I2	wild boar	*Sus scrofa*	-15.0	-13.8	-14.4	1.1	-8.4	-6.4	-7.4	2.0
ALS Sus18 C	wild boar	*Sus scrofa*	-13.9	-11.6	-12.7	2.3	-8.6	-5.6	-7.1	3.0
ALS Sus19 M3	wild boar	*Sus scrofa*	-14.2	-13.6	-13.9	0.6	-9.3	-7.6	-8.4	1.7
ALS Sus21 M3	wild boar	*Sus scrofa*	-13.6	-12.4	-13.0	1.2	-7.4	-6.2	-6.8	1.2
ALS Sus22 M3	dom. pig	*Sus domesticus*	-11.5	-11.0	-11.2	0.5	-6.7	-5.7	-6.2	1.0
ALS Ovis1 M3	sheep	*Ovis aries*	-12.4	-10.7	-11.5	1.6	-7.8	-2.9	-5.3	4.9
ALS Ovis2 M3	sheep	*Ovis aries*	-13.8	-11.4	-12.6	2.5	-9.6	-2.4	-6.0	7.3
ALS Ovis3 M3	sheep	*Ovis aries*	-12.6	-10.1	-11.4	2.6	-8.4	-0.6	-4.5	7.8
ALS Ovis4 M3	sheep	*Ovis aries*	-12.2	-10.7	-11.5	1.5	-6.8	-2.1	-4.4	4.7
ALS Ovis5 M3	sheep	*Ovis aries*	-11.5	-9.7	-10.6	1.8	-7.5	-0.8	-4.1	6.7
ALS Ovis6 M3	sheep	*Ovis aries*	-13.7	-10.6	-12.1	3.0	-7.6	-1.4	-4.5	6.2
ALS Ovis7M3	sheep	*Ovis aries*	-13.2	-10.8	-12.0	2.4	-9.6	-3.7	-6.7	5.9
ALS Ovis8 M3	sheep	*Ovis aries*	-12.9	-11.5	-12.2	1.4	-8.9	-3.5	-6.2	5.4
ALS Ovis9 M3	sheep	*Ovis aries*	-13.6	-11.6	-12.6	2.0	-7.7	-1.7	-4.7	5.9
ALS Bos1 M3	cattle	*Bos taurus*	-11.0	-9.7	-10.3	1.4	-7.0	-2.9	-4.9	4.1
ALS Bos2 M3	cattle	*Bos taurus*	-14.0	-11.8	-12.9	2.2	-7.3	-2.8	-5.0	4.6
ALS Bos3 M3	cattle	*Bos taurus*	-12.6	-10.4	-11.5	2.2	-8.2	-4.1	-6.1	4.1
ALS Bos4 M3	cattle	*Bos taurus*	-11.2	-10.1	-10.6	1.2	-9.1	-1.5	-5.3	7.6
ALS Bos5 M3	cattle	*Bos taurus*	-11.8	-10.2	-11.0	1.6	-8.7	-5.4	-7.1	3.2
ALS Bos6 M3	cattle	*Bos taurus*	-12.0	-10.6	-11.3	1.4	-8.5	-5.6	-7.0	2.9
ALS Bos7 M3	cattle	*Bos taurus*	-11.9	-10.9	-11.4	1.0	-7.8	-3.8	-5.8	4.1
ALS Bos8 M3	cattle	*Bos taurus*	-12.0	-10.0	-11.0	2.0	-6.8	-4.0	-5.4	2.9
ALS Bos9 M3	cattle	*Bos taurus*	-12.6	-10.8	-11.7	1.8	-6.2	-3.7	-5.0	2.5
ALS Bos10 M3	cattle	*Bos taurus*	-13.3	-11.8	-12.5	1.5	-7.0	-3.2	-5.1	3.8
ALS Bos11 M3	cattle	*Bos taurus*	-12.0	-9.9	-11.0	2.1	-7.6	-3.8	-5.7	3.8

For additional information see S2 File. Intra-tooth minimum (Min) and maximum (Max) value;

M = (Min+Max)/2; Δ = Max−Min. Sheep and cattle δ^18^O data were published in [85, 86], respectively.

Cattle enamel bioapatite δ^13^C values vary between −14.0‰ and −9.7‰. All cattle have recorded seasonal variations in their diet δ^13^C values (Fig 4) with amplitudes of intra-tooth variation of 1.0‰ to 2.2‰. As with sheep, most cattle follow Pattern A (ALS Bos4, Bos5, Bos8, Bos9, Bos10 and Bos11) or Pattern B (ALS Bos2 with a winter δ^13^C value of −14.0‰) with phase shifts between the δ^18^O and δ^13^C sequences varying from 310° to 358°; Table H in S2 File). ALS Bos1, Bos6 and Bos7 do not show a sinusoidal pattern of variation in δ^13^C values (Pattern C) but rather have stable values over the summer and the preceding or consecutive winter recorded in the M3 (Fig 4).

The suid teeth have recorded seasonal variations in enamel bioapatite δ^18^O values (Fig 5). Over a year is recorded in the wild boar canine (ALS Sus18 C) and a complete year may be reconstructed when combining the wild boar first and second incisors (ALS Sus20 I1 and I2) whose formations overlap in time [51]. The sequences recorded in the wild boar and pig M3s do not reflect complete annual cycles. Overall, the δ^13^C values recorded in the suids teeth vary between −15.0‰ and −11.1‰. In the wild boar canine (ALS Sus18 C), the δ^13^C values vary in phase with the δ^18^O sequence (phase shift = 355°) with an amplitude of variation of 2.3‰. The pattern of variation in ALS Sus20 I1&I2 also shows a trend for lower δ^13^C values in winter (−15‰) and higher in the summer (−13.1‰). The short δ^18^O sequences recorded in the three suid third molars are centered on the summer; in these teeth, the δ^13^C values vary little around −14‰ and −13‰ in the wild boar M3s (ALS Sus19 and Sus21), although a steep gradient towards higher values (−11.1‰) is measured in the part of the tooth that was formed last, and corresponding to late summer, in ALS Sus21M3 (Fig 5). In the only pig’s molar (ALS Sus22 M3), δ^13^C values vary between −11.5‰ and −11‰.

**Fig 6:**
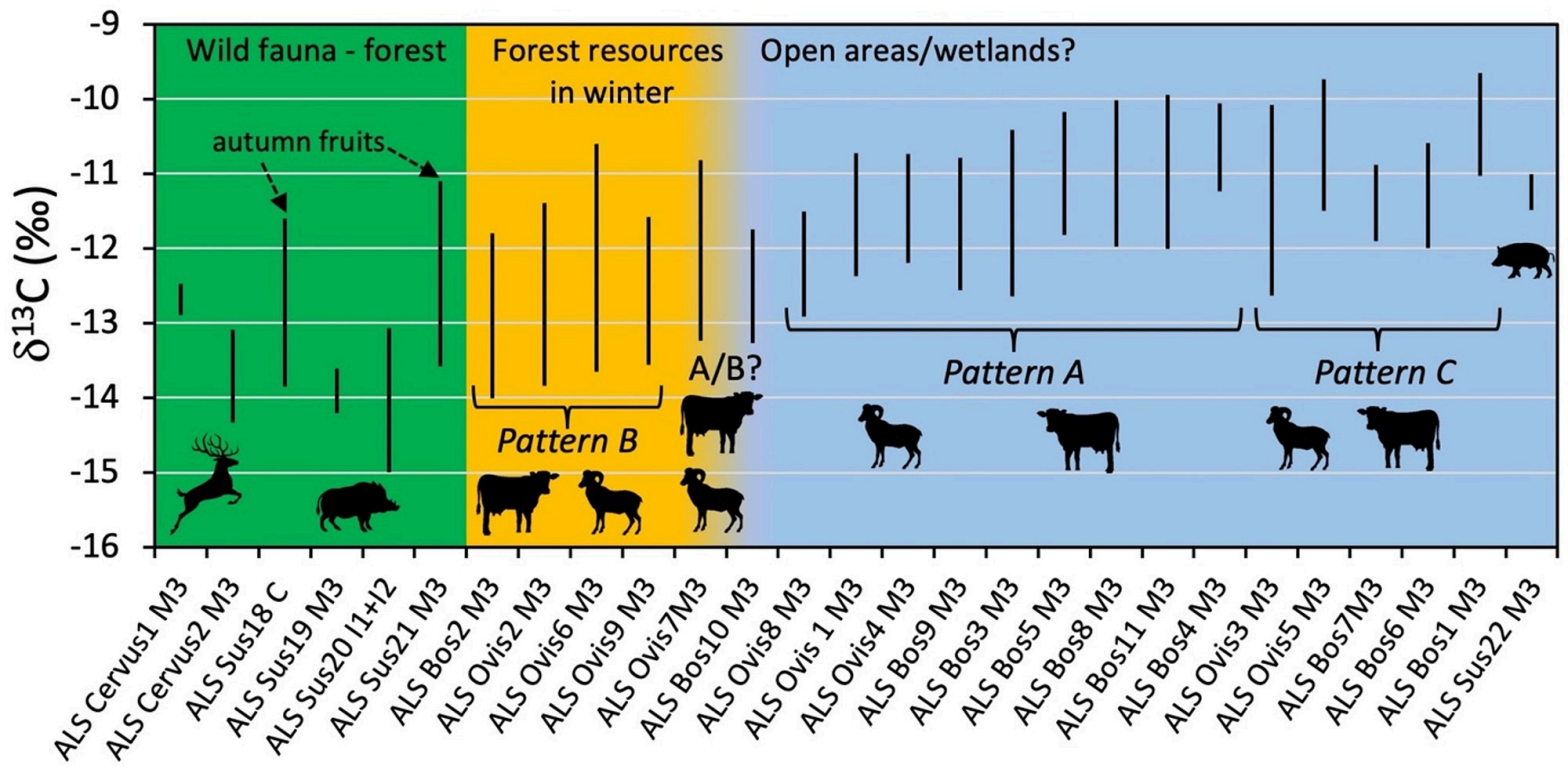
Range of variation of tooth enamel δ^13^C values in the red deer (Cervus), wild boar (Sus18, 19, 20 and 21), cattle (Bos), sheep (Ovis) and pig (Sus22) from the Starčevo phase at Alsónyék. Patterns A, B and C: see main text.

The δ^18^O sequences measured in the two red deer third molars show a steep decreasing trend suggesting a record over a summer-autumn-winter sequence. Over this period, these deer have recorded decreasing δ^13^C values comprised between −12.5‰ and −12.9‰ in ALS Cervus1 and between −13.4‰ and −14.3‰ in ALS Cervus2. Fig 6 compares the range of variation in δ^13^C values in all teeth. The wild fauna (red deer and wild boar) shows the lowest δ^13^C values, in contrast to higher δ^13^C values in domestic animals (pig, sheep, and cattle), although in some of the cattle and sheep teeth, lower δ^13^C values are observed on a seasonal basis.

## Discussion

### Wild fauna: Setting Alsónyék’s surrounding landscape

The bone collagen δ^13^C values of benthic fish were lower and the bone collagen δ^15^N values of the piscivorous fish were more elevated compared to nearly all terrestrial bone collagen samples (Fig 2), revealing a clear separation in stable isotope ratios between animals occupying the terrestrial and aquatic domains. Freshwater fish consumption would therefore lead to higher bone collagen δ^15^N values, as well as lower δ^13^C values, if benthic fish were consumed.

Feeding in forested areas likely explains the lower δ^13^C values in some of the terrestrial animals, with e.g. roe deer ALS Cap9 having the lowest measured δ^13^C_collagen value_ of −23.7‰. Some of the δ^13^C_enamel_ values are similarly low (around −13.1‰ in ALS Cervus1 and as low as −14.3‰ in Cervus2). These low δ^13^C values are consistent with year-round grazing/browsing in a forested area with plants subject to the canopy effect, possibly located on the hills to the west of the site. However, around two thirds of the 19 deer had δ^13^C_collagen_ values between −21.5‰ and −19.8‰, likely reflecting feeding in predominantly open areas.

Measured wild boar collagen δ^13^C values between −21.1‰ and−19.9‰ suggest none of the wild boar sampled for bone collagen fed exclusively on undergrowth of dense forests, and may have moved frequently between forests and more open areas, possibly occupying similar habitats to wild and domestic herbivores. The sequential enamel samples give a more detailed picture: The low δ^13^C_enamel_ values from the four sampled wild boar (Fig 6) indicate feeding in predominantly closed areas, with higher δ^13^C_enamel_ values recorded in late summer/autumn in ALS Sus18C and ALS Sus21M3 (Figs 5 and 6) likely reflecting a greater contribution of forest fruits (e.g. acorns) at this time of the year for these individuals [51, 87].

The wild boar collagen δ^15^N values showed very wide variability, from 4.0‰ to 8.6‰ (cf. a biological variability of ca. 1.5‰ for domestic pigs of differing ages and sex when consuming the identical diets; [45]). This suggests large differences in trophic levels between different wild boar individuals. Two individuals had similar δ^15^N values to dogs (> 8‰: ALSSuss6, ALSSuss12), suggesting a large degree of animal protein consumption, possibly partly by feeding on wetland fish naturally trapped after flooding [88]. In the case of wild boars with low δ^13^C and high δ^15^N values, mushroom consumption may also have elevated the δ^15^N values [89]. This large variability in the wild boar baseline δ^15^N values complicates the interpretation of domestic pig diets.

### Cattle and sheep husbandry

The cattle and sheep collagen δ^13^C values suggest grazing in predominantly open areas, possibly also including wetlands. Ethnographic evidence from the Carpathian basin describes wetlands being used as pastures [90–92], although mainly for cattle and pigs. Historical accounts from Hungary from the 18^th^ to 20^th^ centuries describe wetlands being seen as dangerous for sheep because they were liable to catch parasites (liver fluke), from which they could not be cured [93]. Floodplains and marsh edges were also described as having a predominance of unappealing, less nutritious grasses, which were still used as reserve pastures, particularly in dry years [93, 94]. Keeping sheep in the flat wetlands despite the disadvantageous environmental conditions has been suggested from lesions found on sheep remains from Körös settlements in eastern Hungary [95–97]. Nevertheless, caprine remains rather than pig remains are dominant in the faunal assemblages [96]. Cultivated fields could also have been used for grazing on a seasonal basis. In five of the twenty analysed sheep and cattle molars, the sequential enamel δ^13^C values deviate from the expected sinusoidal pattern of variation with a reduced amplitude of variation between δ^13^C values recorded in winter and the consecutive or preceding summer (Pattern C, Fig 4 and S2 File). In sheep (ALS Ovis3, Ovis5), this reduced amplitude appears to be due to a rise in the winter δ^13^C values, possibly caused by summer hay being provided in winter. Such hay could have consisted of crop wastes and grass (including wetland grasses; [93, 94]). In some cattle, the reduced amplitude of variation might rather be due to a lowering in summer δ^13^C (ALS Bos 1 and Bos6), possibly caused by forest leaves provided in the summer. Ethnographic accounts report that tree-fodder can be particularly important for cattle and sheep in dry summers [93, 98, 99].

Forest resources also seem to have been used to supplement cattle and sheep diets during winter, as evidenced in four of the twenty analysed sheep and cattle molars showing significantly lower δ^13^C values in winter (Pattern B, Figs 4 and 6). The phase shift between δ^13^C and δ^18^O sequences does not deviate from what is observed in other specimens, meaning that this contribution occurs mainly when temperatures are the lowest in winter. In the Alsónyék area of Hungary in the 20^th^ century, snow cover in winter lasted an average of 35–40 days [100], reaching up to 25 cm [18]. Similar conditions appear to have prevailed in the past (within 1 °C of modern values; [101]), limiting the animals’ foraging activities in winter. Ethnographic evidence from Central Europe in the 18^th^ to 20^th^ century indicates that domesticated animals were frequently kept in deciduous forests from spring until heavy snowfall in winter. Historical accounts from Hungary describe winter fodder being prepared by collecting and drying leaf-bearing twigs and branches, mistletoe, as well as acorns and beechnuts [93]. Most other sheep and four cattle had winter δ^13^C_enamel_ values between −12‰ and −13‰, indicating that these individuals could also have consumed forest resources with low δ^13^C values, but to a lower extent (or possibly in combination with larger amounts of acorns, which are higher in δ^13^C), or may have relied on freshwater wetland grasses.

There is little indication of forest resource use in the bone collagen δ^13^C values, apparently contrasting with the enamel results. This discrepancy is likely due to the averaging effects in bone, whereby the effects of short-term consumption of foods with different δ^13^C values are attenuated [102].

### Pig husbandry

Historic sources from the 18^th^ to 20^th^ centuries show that pigs frequently had very varied diets, for example feeding in wetlands, where they were able to eat the vegetation, bird eggs and fish [93], and were frequently freely ranging in oak forests in autumn until they could not find acorns any more in winter and returned to where they were fed by humans [93].

The wide range of measured bone collagen δ^15^N and δ^13^C values shows large variability in pig diets. Eleven of the sampled pigs from Alsónyék had low δ^15^N values, between 6.1‰ and 7.7‰, similar to most of the sampled wild boar, with δ^13^C values mostly above −21.5‰ suggesting primarily herbivorous feeding in a predominantly open environment. However, the consumption of acorns (elevated δ^13^C values) may mask feeding on forest undergrowth [51]. The high enamel δ^13^C values (−11.5‰ to −11.0‰) for the sampled pig molar suggest feeding in an open environment for this individual. Since current ecological studies have shown wild boar diets to generally consist of around 86–96% plant matter (reviewed in [68]), and because the δ^15^N values of wild and domesticated herbivores overlap with those of pigs, a highly plant-based diet can be inferred from these results for most of these domestic pigs.

Suids likely thrived in the environment surrounding Alsónyék, where acorn-bearing forests met with wet, marshy areas (cf. [23, 93]), so it is notable that pigs only made up 3% (NISP_identifiable mammal_) of the mammal assemblage recovered from the Starčevo phase at Alsónyék [23]. This is comparable to the Starčevo site Lánycsók-Égettmalom and the Körös/Criş sites Ecsegfalva 23 and Endrőd 119, all in Hungary (Fig 1), where similarly wet conditions likely conducive to pig-keeping were present, but only low numbers of pigs were recovered (0.6% to 3% of animal remains by NISP_total_; [95, 103–105]). This scarcity of pigs is also visible in the scarcity of non-ruminant adipose fats revealed by residue analysis of pottery sherds from both Alsónyék and Ecsegfalva 23 [106]. The near absence of pigs in favour of caprines and cattle has been suggested to be due to culturally driven motivations, possibly related to emotional reasons or taste preferences at other early Neolithic sites in eastern Hungary [94, 95].

**Fig 7:**
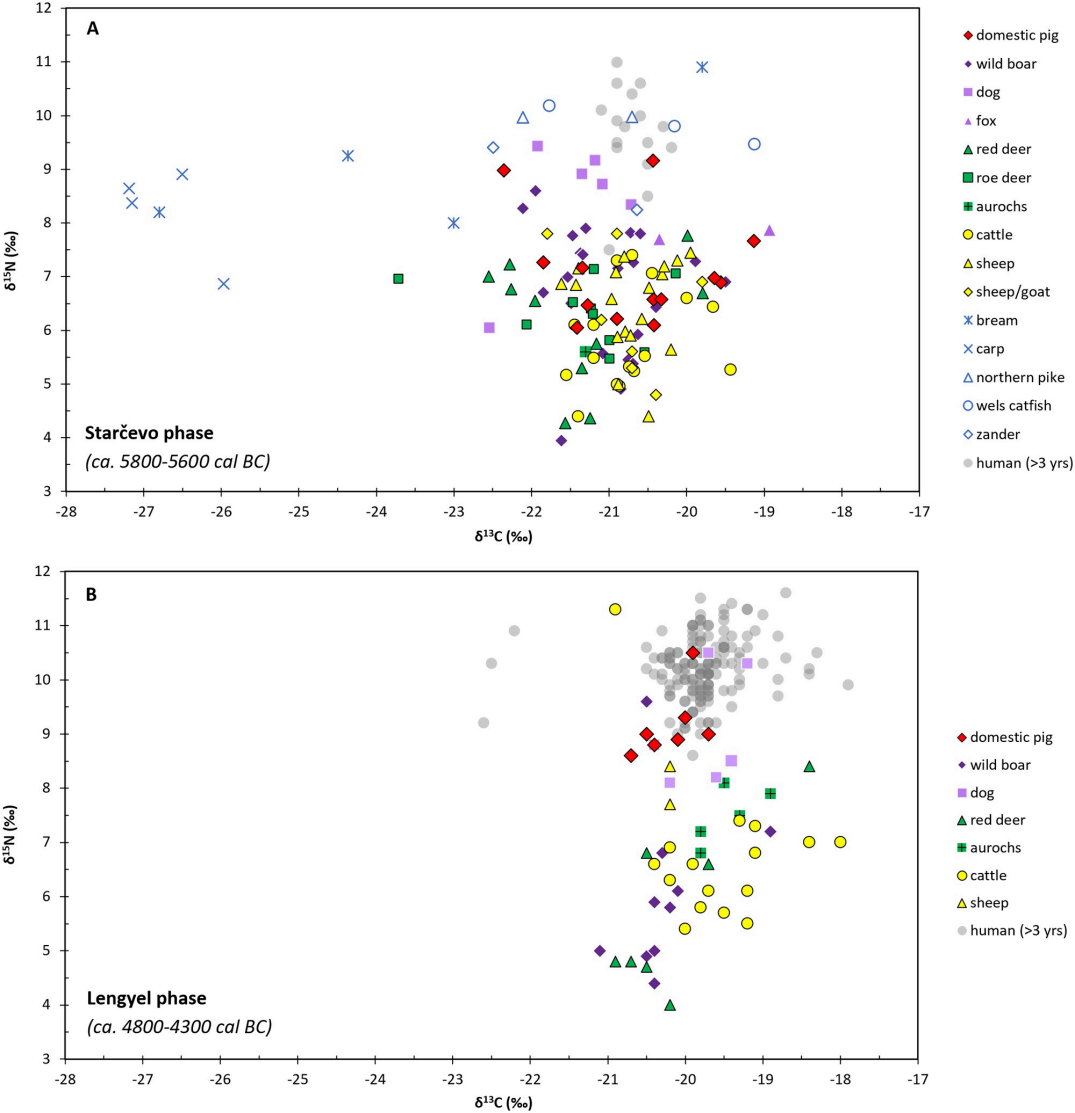
Stable carbon (δ^13^C) and nitrogen (δ^15^N) isotope ratio results for bone collagen from Alsónyék. (A) Starčevo phase, data from this study and [25]. (B): Lengyel phase, data from [25].

Two of the 13 analysed pigs (ALS Susd5 and Susd8) had elevated δ^15^N values of 9.0‰ and 9.2‰, similar to dogs and the lower end of the spectrum of human δ^15^N values (Fig 7A; [25]), indicating consumption of fertilised crops or higher trophic level foods. Historical evidence from Hungary suggests these could have been, for example, meat, freshwater fish (stable isotope ratios suggest pike, catfish and zander in the case of dogs and ALS Susd5), whey, excess milk and leftover human food [93].

Ethnographic evidence from Greece shows that household pigs (i.e. 1–2 pigs stall-fed by one or more households) tended to be given dairying by-products, kitchen scraps and surplus products, whereas larger herds were taken to forage in fields and woods, and fed cereals in dry summers, or to encourage their return [107]. The bone collagen data from the Starčevo phase Alsónyék therefore suggest that a small number of pigs may have been kept as household pigs, intensely fed, whereas most pigs were kept extensively, grazing and foraging for themselves, with occasional dietary supplementation by humans.

### Comparison to other phases at Alsónyék

**Fig 8:**
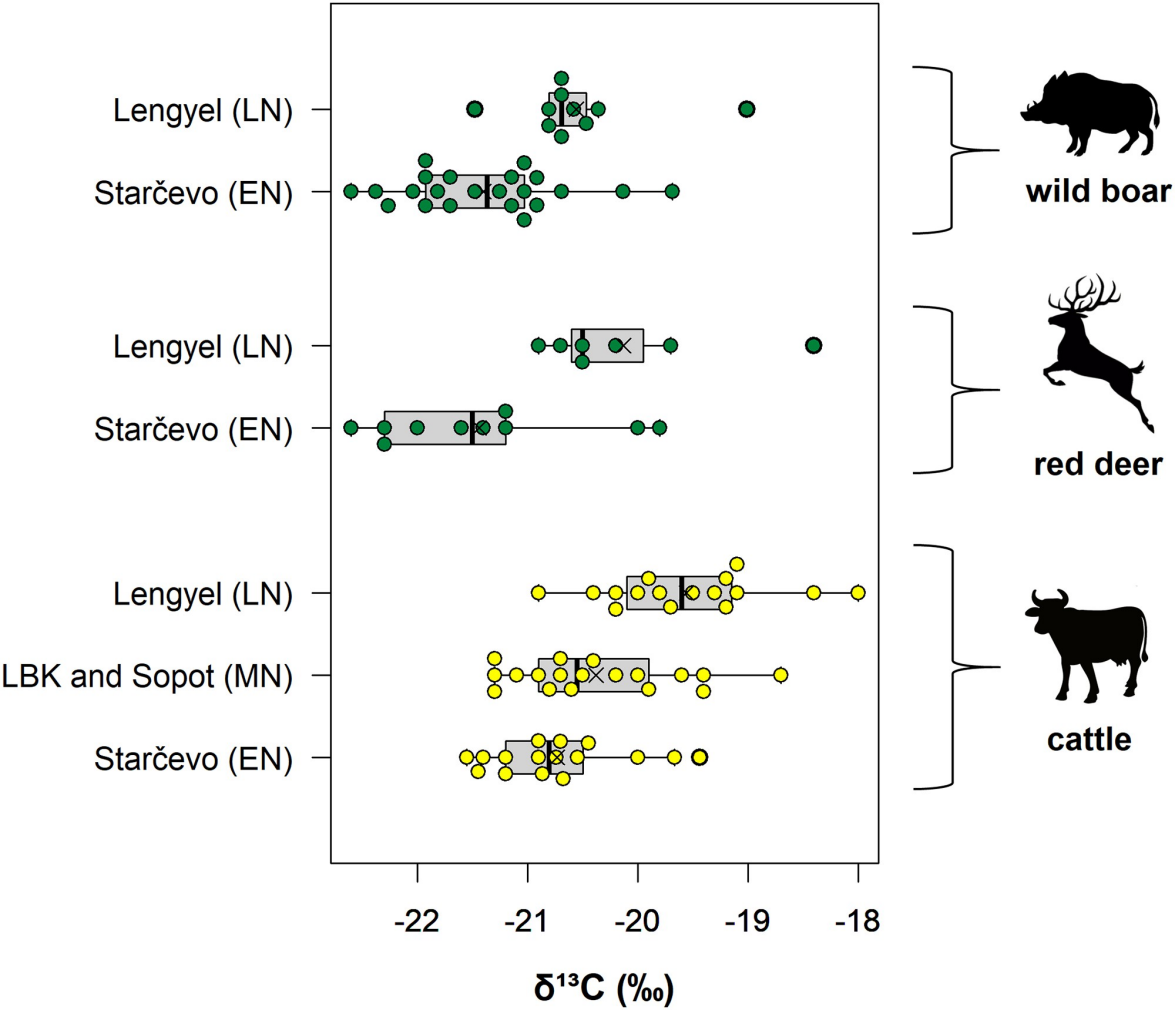
Stable carbon (δ^13^C) isotope ratio results for red deer, wild boar and cattle bone collagen from the Starčevo, LBK, Sopot and Lengyel phases at Alsónyék, shown as boxplots. The line in the boxplot indicates the median, and the cross the mean. Whisker boundaries were chosen as 1.5 × interquartile range. Data from this study and [25].

As part of earlier work, faunal and human remains from Starčevo, Linearbandkeramik (LBK), Sopot and Lengyel phases (see Table 1; [14]) at Alsónyék were analysed for bone collagen δ^13^C and δ^15^N [25]. Comparison to this study indicates cattle, red deer and wild boar δ^13^C values becoming higher in later phases at Alsónyék (Fig 8). This suggests feeding in more open areas and less reliance on forest resources after the Starčevo phase. As this trend appears not only for cattle (domesticated animals), but also for wild boar and red deer (wild animals), this suggests deforestation from the Starčevo phase towards the Lengyel phase, likely as a result of increased grazing pressure, cutting down trees for wood, and/or intentional forest clearing.

Pigs from the Lengyel phase at Alsónyék had ca. 2–3 ‰ higher δ^15^N values than wild boar, cattle and sheep (Fig 7B), suggesting better access to animal protein (or fertilised crops). Pig remains were around three times as common (as a proportion of domestic animals) at Alsónyék during the Lengyel period compared to the Starčevo period [22], which, coupled with the difference in pig diets, could indicate a higher importance placed on pig keeping by the Lengyel than by Starčevo people at Alsónyék.

### Comparison to other sites

To place the above results into a broader context, the literature was reviewed to identify numerically published faunal bone collagen δ^13^C and δ^15^N datasets with n_faunal_≥25 and n_pigs_≥5 from Hungary, Greece, Croatia, Serbia and Romania from the Early Neolithic to the Chalcolithic (Fig 1). Where data from multiple Neolithic phases was available, the earliest phase was compared to (cf. Table 1).

Analysis of remains from LBK phases at Füzesabony, Hungary, and from the Vinča phase at Stubline, Serbia, showed a large overlap between herbivore and pig δ^13^C and δ^15^N values [26, 27], suggesting pigs had little or no access to animal protein (e.g. whey, slaughter waste). (This trend is also consistent with data from Măgura—Boldul lui Moş Ivčnus, but only two domestic pigs have been analysed from this site so far [33]). By comparison, Early Neolithic remains from Zemunica, Croatia, show several pigs had low δ^13^C and δ^15^N values compared to cattle and sheep/goats [34], suggesting these pigs were kept primarily on forest resources, while cattle and sheep were less reliant on forest resources. At Zemunica, one analysed outlier individual had higher δ^15^N values, and may have been a stall-fed pig [34].

Remains from Vinča-Belo Brdo (see Table 1 and Fig 1; [27]), Halai [31], Kouphovouno [108], Makriyalos [31, 109] and Balatonszárszó-Kis-erdei-dűlő [26] all showed an overlap between pig, cattle and sheep δ^13^C and δ^15^N values. However, at these sites, there was a tendency for pig δ^15^N values to be on average slightly elevated compared to sheep and cattle, while pigs with δ^15^N values toward the lower end of the spectrum displayed by cattle and sheep are missing. This suggests some (limited) availability of animal protein to pigs. Additionally, several outlier pigs at these sites had elevated δ^15^N values, similar to the results seen for the Starčevo phase at Alsónyék, which appear to have had more access to animal protein (or fertilised crops), possibly as a result of being more intensely kept household pigs. From these data, a picture seems to be emerging, whereby pig husbandry strategies employed in Southeast Europe (cf. Fig 1) during the Early and Middle Neolithic mostly involved extensive pig herding (with occasional dietary supplementation) for the majority of individuals, while a few pigs were more intensively kept, possibly stall-fed in villages for at least part of their lives. Future data will confirm or deny this apparent trend.

In contrast, results from the late Chalcolithic (second half of 5^th^ millennium BC) sites Vităneşti-Măgurice, Hârşova-tell and Borduşani-Popină, all in southeastern Romania, show only little overlap in δ^15^N values between pigs and herbivores, suggesting pig diets at these sites to have comprised significantly larger amounts of animal protein [28]. Therefore, there appear to be clear spatial and temporal trends in pig keeping, the underlying affecting factors for which (whether environmental, cultural or other) remain to be discovered.

## Conclusion

The results of this study showed a large extent of overlap in bone collagen δ^13^C and δ^15^N values for pigs, wild boar, domesticated and wild herbivores from the Starčevo phase at Alsónyék, suggesting that these animals may well have shared the same grazing/browsing areas for at least parts of the year. The spread of the data demonstrates variability between individuals of the same species, indicating that on an individual basis, different foods and habitats were used to different extents. Dense woodland or open environment “specialists” were the exception, and most of the studied animals from the Starčevo phase appear to have made use of the variability of diverse resources provided by the environment surrounding Alsónyék. The differences between individual cattle, sheep and pig diets could be due to metabolic variation, interannual variability in weather conditions, differing animal husbandry practices between households, and specific individuals being chosen to receive special treatment.

The collagen and enamel δ^13^C data from the Starčevo phase at Alsónyék indicate that sheep and cattle tend to have fed in more open areas dominated by C_3_ plants (possibly including grazing stubbles on croplands) compared to some of the roe deer and red deer. Sequential enamel analyses showed seasonal variability in the diets of sheep and cattle, whereby winter diets consisted of either grazing in open environments, consumption of forest resources, provision with summer hay, or a combination thereof. This variability in winter diets of domesticated herbivores suggests multiple strategies for coping with the challenges of wintery conditions, made feasible by the diverse environment around Alsónyék.

Most pigs appear to have mainly consumed plant matter, which, combined with the low numbers of pig remains at Alsónyék during the Starčevo phase, suggests pig husbandry at only very low intensity. Only a few pigs appear to have received greater amounts of dietary supplementation with animal protein or fertilised crops, emphasising the non-intensive character of most pig husbandry at Alsónyék during the Early Neolithic Starčevo phase.

Comparisons of the data in this study with published datasets from Neolithic sites in Greece, Serbia, Croatia and Hungary suggests that the large extent of overlap in bone collagen δ^13^C and δ^15^N values for pigs, wild boar, domesticated and wild herbivores observed in this study was not uncommon in this period. Sharing of the same grazing/browsing areas by different wild and domestic species may have been widely practiced during the 6^th^ and 5^th^ millennium in Southeast Europe.

The comparison of our data with those from later assemblages at the same site indicates a trend to higher δ^13^C values for cattle, red deer and wild boar during the later phases at Alsónyék (Fig 8). This suggests a shift to less reliance on forest resources after the Starčevo phase, likely due to increased deforestation. These observations reinforce the importance of using animals from the same period as baselines for studies of human diets. Thus, in addition to elucidating past dietary management of domestic stock at Alsónyék, this study also provides baseline δ^13^C and δ^15^N values for future animal and human isotope ratio studies of Early Neolithic people in the Carpathian Basin.

## Supporting information

S1 File Bone collagen δ^13^C and δ^15^N results. Contains Tables A and B.
(XLSX)

S2 File Tooth enamel δ^13^C and δ^18^O results. Contains Tables C-H.
(XLSX)

S3 File Figures of bone collagen δ^13^C and δ^15^N results. Includes brief explanation of Bayesian ellipse modelling.
(PDF)
