# Supplementary material for: Early Neolithic pastoral land use at Alsónyék-Bátaszék, Hungary (Starčevo culture): New insights from stable isotope ratios
Source: PLoS One. 2023 Dec 12;18(12):e0295769. doi: 10.1371/journal.pone.0295769 (PMC10715649; doi:10.1371/journal.pone.0295769)
Supplement: S3 File — Includes brief explanation of Bayesian ellipse modelling. (PDF) [file pone.0295769.s003.pdf]

# S3 File: Figures of bone collagen δ$^{13}$C and δ$^{15}$N results

The ellipses in Figs 2 and 2a are Bayesian ellipses, created using the R package SIBER. They are created to have a 90% probability of containing a subsequently sampled datum from the same population. In Fig 2 an outlier dog and two pigs (identified as outliers by hierarchical cluster analysis) were manually excluded prior to creating the ellipses. Fig 2a shows ellipses for all data, including outliers. Fig 2b shows the same data with convex hulls instead of ellipses.

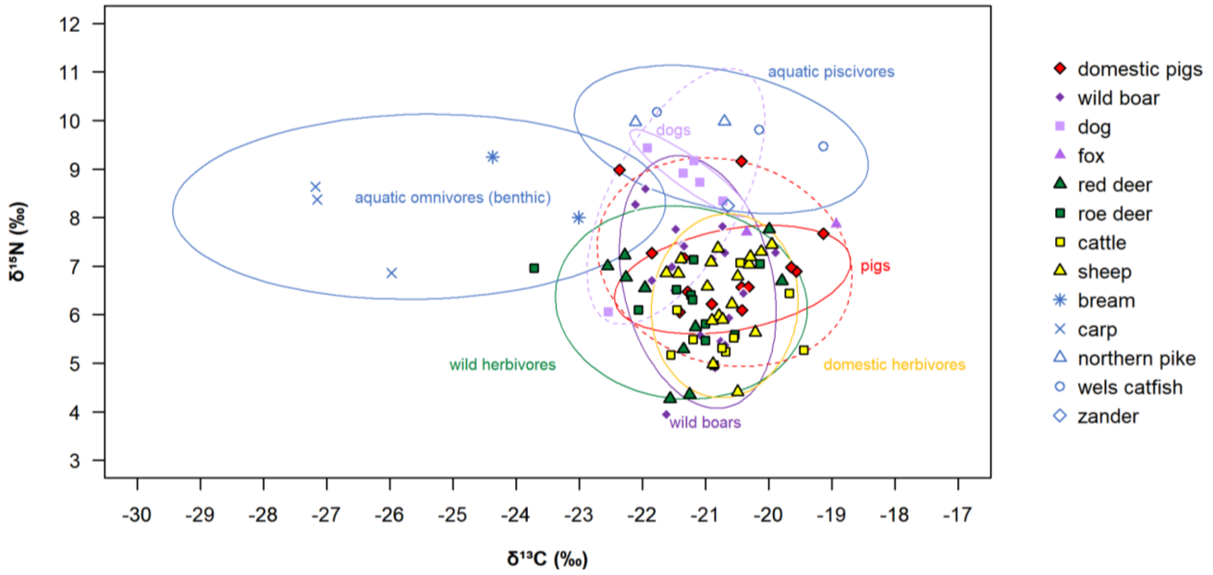


**S3 File Fig. 2a Stable carbon (δ$^{13}$C) and nitrogen (δ$^{15}$N) isotope ratio results for bone collagen from the Starčevo phase at Alsónyék, subsite 5603/1.** The 90% prediction ellipses were modelled excluding an outlier dog and two pig datapoints (solid lines), and including all data (dashed lines in the case of pigs and dogs).

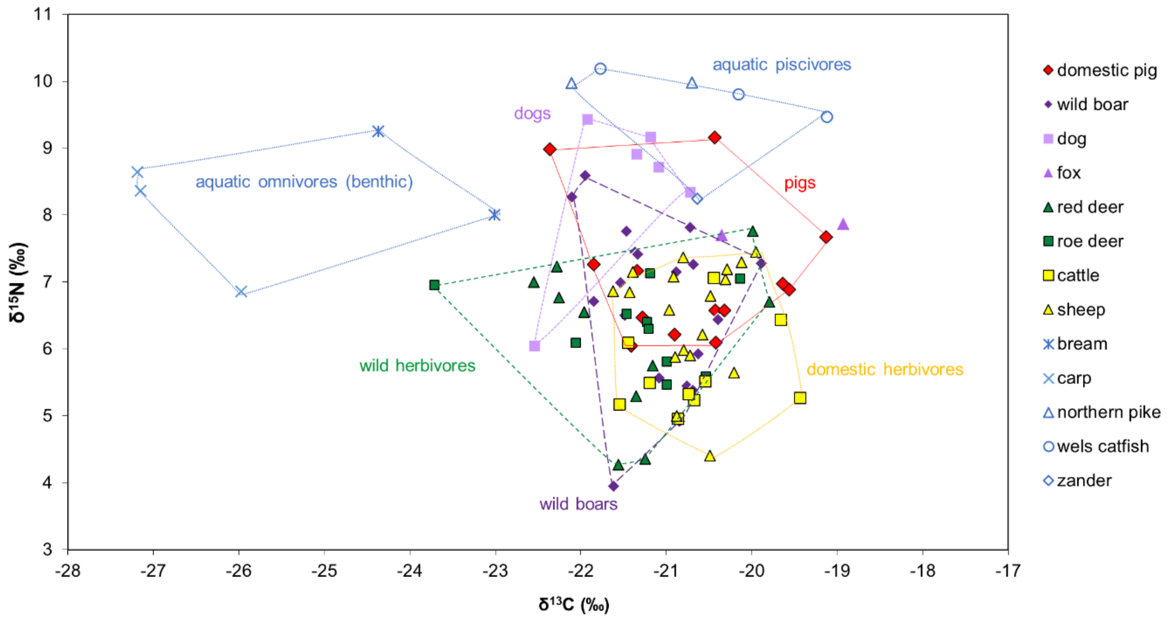


**S3 File Fig. 2b Stable carbon (δ$^{13}$C) and nitrogen (δ$^{15}$N) isotope ratio results for bone collagen from the Starčevo phase at Alsónyék, subsite 5603/1.** Depiction using convex hulls.
